# Comparison of Allotransplantation of Fresh and Vitrified Mouse Ovaries to The Testicular Tissue under Influence of The Static Magnetic Field 

**DOI:** 10.22074/cellj.2017.4513

**Published:** 2017-08-19

**Authors:** Vida Sadat Kazemein Jasemi, Firooz Samadi, Hussein Eimani, Saeed Hasani, Rouhollah Fathi, Abdolhossein Shahverdi

**Affiliations:** 1 Department of Animal and Poultry Physiology, Faculty of Animal Science, Gorgan University of Agricultural Sciences and Natural Resources, Gorgan, Golestan, Iran; 2 Department of Embryology, Reproductive Biomedicine Research Center, Royan Institute for Reproductive Biomedicine, ACECR, Tehran, Iran; 3 Department of Anatomy, Faculty of Medicine, Baqiyatallah University of Medical Sciences, Tehran, Iran

**Keywords:** Apoptosis, Magnetic Field, Mice, Transplantation, Vitrification

## Abstract

**Objective:**

The aim of this study was to investigate the effects of static magnetic field (SMF) during transplantation of the ovarian tissue into the testis.

**Materials and Methods:**

In this experimental study, ovaries of 6- to 8-week-old female Naval Medical Research Institute (NMRI) mice were randomly divided into four groups: i. Fresh ovaries were immediately transplanted into the testicular tissue (FOT group), ii. Fresh ovaries were exposed to the SMF for 10 minutes and then transplanted into the testicular tissue (FOT+group), iii. Vitrified-warmed ovaries were transplanted into the testicular tissue (VOT group), and iv. Vitrified-warmed ovaries were transplanted into the testicular tissue and the transplantation site was then exposed to the SMF for 10 minutes (VOT+group).

**Results:**

The lowest percentages of morphologically dead primordial follicles and the highest percentages of morphologically intact primordial follicles were seen in the FOT+ group (4.11% ± 2.88 and 41.26% ± 0.54, respectively). Although the lowest significant percentage of maturation, embryonic development and fertility was observed in the VOT group as compared to the other groups, the difference in the fertility rate was not significant between the VOT and VOT+groups. Estrogen and progesterone concentrations were significantly higher in the FOT+group than those of the control mice.

**Conclusion:**

It is concluded that, exposure of the vitrified-warmed ovaries to SMF retains the structure of the graft similar to that of fresh ovaries.

## Introduction

Ovarian tissue transplantation is an option for fertility preservation. Usually, ovarian tissue transplantation is performed by re-implanting the tissue in the body of donor animal (autograft), to a recipient of the same species as donor (allograft) or to a recipient from a species different from the donor (xenograft) (1). Ovarian tissue pieces can be grafted to their original location (such as the ovarian cortex and the peritoneum under the ovary hilus) (2, 3), which would allow pregnancy devoid of further medical assistance (4), or in other places (for example, the right abdominal muscle, kidney and uterus) (5, 6). Ovarian transplantation into the testis was first done by Sand (7), which resulted in growth of follicles until maturation stage. Takewaki (8) also reported the survival of the ovarian tissue transplanted into the testis for up to 2 months. Ovarian transplantation into the testis may be a unique technique for assessing the developmental potential of the ovaries in a situation where the levels of folliclestimulating hormone and luteinizing hormone are significantly decreased, and may also be useful for saving ovaries with genetic defects. However, little information is available about using the testicular tissue as a transplantation site.

The key challenges dealing with transplantation are tissue ischemia and delayed vascular anastomosis after transplantation (9). Ischemic damages and lack of blood flow to the tissue, which leads to defects in the delivery of oxygen and nutrients to the tissues, may destroy up to 70% of the primordial follicles (10). To solve this problem, many researchers have attempted to minimise the length of time that the tissue must spend in an ischemic state after ovarian transplantation. Some studies have shown that magnetic field can help prevent or repair damage caused by ischemia, and may also be effective in reducing the occurrence of the apoptosis (programmed cell death) and reestablishing blood flow (11, 12). Therefore, the purpose of the present study was to investigate the effects of static magnetic field (SMF) on fresh and vitrified ovarian tissues transplanted into the testis.

## Materials and Methods

### Preparation of mouse ovary and experimental design

The study was approved by the Ethics Committee of Royan Institute, Tehran, Iran. In this experimental study, sixty 6- to 8-weekold female Naval Medical Research Institute (NMRI) mice weighing 20 to 30 g were obtained from the animal house of Royan Institute (Tehran, Iran). They were kept at an appropriate condition (18-22˚C, 12/12- hours light/dark cycle) with free access to food and water. In each repetition, the ovaries were removed from the body and randomly divided into four groups (fifteen ovaries in each group): i. Fresh ovaries were immediately transplanted into the testicular tissue (FOT group), ii. Fresh ovaries were exposed to a magnetic field for 10 minutes and then transplanted into the testicular tissue (FOT+ group), iii. Vitrified-warmed ovaries were transplanted into the testicular tissue (VOT group), and iv. Vitrified-warmed ovaries were transplanted into the testicular tissue and the transplantation site was then exposed to a magnetic field for 10 minutes (VOT+ group). The magnetic field strength in all cases was 1 millitesla (mT).

### Static magnetic field production

**Fig.1:**
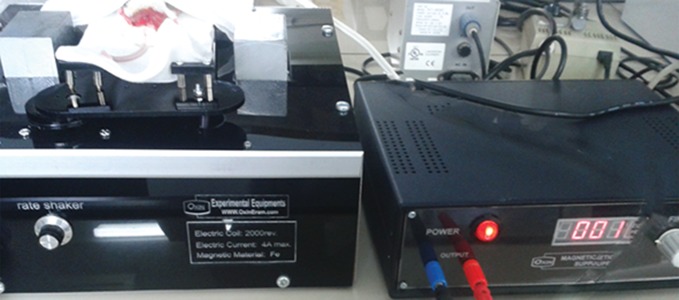
Image of the static magnetic field generating system. Vitrified-warmed ovaries were transplanted into the testicular tissue and the transplantation site was then exposed to a magnetic field for 10 minutes (VOT+ group).

In the present study, an electromagnetic device capable of producing a constant and uniform magnet field of around 1 mT (Fig .1) was used. The magnetic field was generated using two poles of ferrite core, wrapped with 2,000 turn copper wire. The input power of the device was 220 volt AC (50 Hz) which was converted to 4 Ampere (A) direct current for the wire coil to generate a uniform magnetic flux between the poles.

### Vitrification and warming processes

The samples in experimental groups 3 and 4 were vitrified using Behbahanian’s vitrification method with some modification (13). Briefly, the ovaries were transferred into an equilibration medium for 15 minutes at room temperature under the influence of magnetic field. The ovaries were then immediately immersed in a vitrification medium for 30 minutes at 4˚C. After dehydration, each ovary was plunged into liquid nitrogen by using a cryopin, a needle of an insulin syringe (14). For warming, the ovaries were transferred to a warming medium for 10 minutes at room temperature. All media used were prepared according to Behbahanian et al. (13).

### Ovarian transplantation into the testicular tissue

Sixty ovaries of female mice were transplanted into 6- to 8-week-old NMRI male mice that were not genetically identical. Briefly, after the male mouse was anaesthetised (15), a gap with length of approximately 1 cm was created in the abdominal area, and the testis was exposed for further manipulation. Subsequently, four-fifths of seminiferous tubules were drawn away by suction using a 20-gauge needle attached to a 20-mL plastic syringe. The ovary was inserted into the testis with sterile forceps. After transplantation, the hole in the testis capsule was closed by suture, and the testis was returned to its original location in the body (16). The mice were allowed to live for 3 weeks.

### Morphological evaluations and immunohistochemistry study

Three weeks after transplantation, 20 ovaries from recipient testicles in four separate experiment groups were removed. After casting in paraffin, the ovaries were serially sectioned into 6 μm thickness and stained with haematoxylin and eosin. All stained ovarian sections were evaluated, and the follicles were counted by a light microscope (magnification of ×400). To prevent miscalculating or counting the follicles more than once, only those with an observable nucleus of oocytes were counted. Follicles with pyknotic oocyte nucleus, shriveled ooplasm or disorganized granulosa cells were regarded as dead follicles (17). The number of intact and dead follicles was counted according to the study by Liu et al. (6).

Anti-CD31 antibody (Ab28364, 1:100 dilution) was tested to evaluate neo-vascularisation of the grafted tissues. The liver tissue was used as positive and negative controls for CD-31. Angiogenesis was studied by counting and averaging blood vessels using high-power field magnification (×400) in three randomly selected fields per sample. Immunohistochemical study was performed for antiactive Caspase-3 antibody (Abcam, primary antibody ab4051 and secondary antibody ab97051) according to a protocol by Gao et al. (18). The thymus tissue was used as positive and negative controls for caspase-3. In this study, the follicles that contained caspase-3- positive oocytes or more than 30% granulosa cells were considered to be apoptotic follicles. In order to quantify, 10 sections of each ovary was subjected to immunohistochemistry.

### Hormonal assays

For the next 21 days after transplantation, blood samples were retrieved from the heart, and the concentrations of estrogen (E2), progesterone (P4) and testosterone (T4) were measured using a chemiluminescence immunoassay analyser (Roche Diagnostics, GmbH, Germany). Blood samples were collected from four mice in each experimental group. Blood samples were also collected from five non-grafted male mice agematched with the transplanted animals to be used as control sample.

### Gonadotropin treatment and graft recovery

Follicular stimulation was carried out by injection of pregnant mare’s serum gonadotropin (Folligon, Intervet, Castle Hill, NSW, Australia) and human chorionic gonadotropin (hCG, Chorulon, Intervet) hormones (19). Nineteen days after transplantation, each mouse was administered intraperitoneally a dose of 7.5 IU pregnant mare’s serum gonadotropin and a dose of 7.5 IU hCG 48 hours later. Twelve hours after hCG injection, the mouse was killed by cervical dislocation, and the ovary was removed from the male mouse for oocyte isolation.

### *In vitro* maturation of oocytes

The ovaries (n=40) were mechanically dissected using an insulin syringe needle in Alpha Modification of Minimum Essential Medium Eagle (α-MEM) droplets supplemented with 10% v/v fetal bovine serum (FBS) and antibiotic solution (100 U/mL penicillin G and 100 mg/mL streptomycin sulphate). To cultivate oocytes, a solution containing α-MEM medium supplemented with 100 mIU/mL recombinant human folliclestimulating hormone (rhFSH, Gonal-f, Serono), 7.5 IU/mL hCG (Pregnyl, Organon) and 5% FBS was used. After 16 hours of incubation, oocytes with first polar body (as metaphase II) were picked and transferred to a fertilization environment.

### *In vitro* fertilization of oocytes and embryo development

Approximately 7 to 10 of the metaphase II (MII) mature oocytes were added to 100- to 150-μL droplets of sperm suspension with a concentration of 0.8×10^6^ sperm per mL and then incubated for at least 4 hours. After incubation, fused sperms were separated from the oocytes with pipetting, and oocytes with a released second polar body or two pronuclei (2PN) were considered as fertilised oocytes. 2PN embryos were transferred into T6 medium droplets supplemented with 4 mg/mL bovine serum albumin. The cultured embryos were checked and observed at 24, 48, 72 and 96 hours after fertilization.

### Analysis of the data

SPSS 18.0 software (SPSS, Chicago, IL, USA) was used for statistical analysis. The number of morphologically intact, dead and apoptotic follicles in all experimental groups was compared by one-way analysis of variance and Duncan’s test. P<0.05 was considered to be statistically significant.

## Results

### Histological analysis

**Fig.2:**
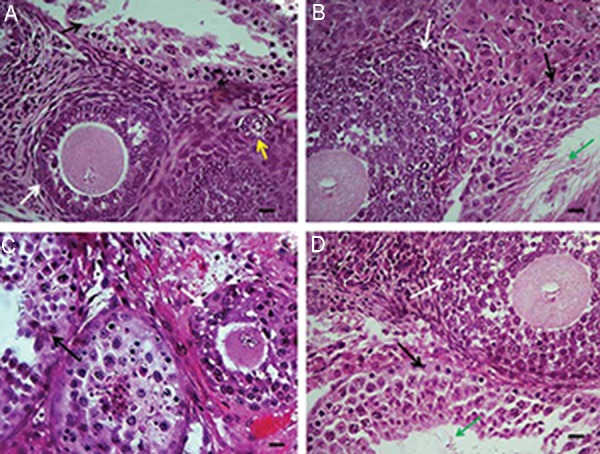
Morphology of mouse ovarian histological sections transplanted into the testis. A. Fresh ovaries were immediately transplanted into the testicular tissue (FOT group), B. Fresh ovaries were exposed to the magnetic field for 10 minutes and then transplanted into the testicular tissue (FOT+ group), C. Vitrified-warmed ovaries were transplanted into the testicular tissue (VOT group), and D. Vitrifiedwarmed ovaries were transplanted into the testicular tissue and the transplantation site was then exposed to a magnetic field for 10 minutes (VOT+ group). White arrow indicates preantral follicle. Yellow arrow indicates primary follicle. T indicates the host tissue (testis). Green arrow indicates the host tissue spermatozoa (scale bars: 10 μm).

There were considerable differences in morphologically intact and dead primordial follicles between the FOT+ group and other groups (Tables1, 2). The mean percentages of morphologically intact primordial follicles in the FOT+ (41.26% ± 0.54) group was significantly higher than those in the FOT, VOT and VOT+ groups (34.88% ± 2.04, 24.82% ± 1.03 and 30.48% ± 1.38, respectively). Furthermore, the VOT group had the lowest percentage of intact primordial follicles (24.82% ± 1.03). The mean percentage of dead primordial follicles in the FOT+ group (4.11% ± 2.88) showed the highest preservation of small follicles, and this was comparable to the FOT (12.88% ± 4.14) and VOT+ (12.33% ± 2.74) groups. There were no significant differences in intact primary follicles between all the groups. Dead primary follicles had a pattern similar to that of intact primary follicles, except for the VOT group (17.24% ± 2.43) in which the largest number of dead primary follicles was seen. In addition, there was a comparative difference in morphologically intact and dead preantral follicles between the FOT (4.87% ± 0.58 and 3.31% ± 2.55, respectively) and VOT (7.67% ± 0.87 and 6.30% ± 2.78, respectively) groups, but the difference was not significant when compared with the FOT+ group (6.23% ± 1.20 and 3.99% ± 2.44, respectively) (Fig .2). The mean percentages of intact and dead antral follicles were not significantly different in all groups (Tables1, 2).

**Table 1:** Number of morphologically intact follicles (mean ± SEM) at different developmental stages 21 days after transplantation


Experimental group n=5	Primordial folliclen (Mean% ± SEM)	Primary folliclen (Mean% ± SEM)	Preantral folliclen (Mean% ± SEM)	Antral folliclen (Mean% ± SEM)	

FOT	436 (34.8 ± 2.04)^b^	352 (28.1 ± 1.58)	61 (4.8 ± 0.58)^c^	21 (1.7 ± 0.25)	
FOT^+ ^	475 (41.3 ± 0.54)^a^	334 (28.9 ± 0.54)	70 (6.2 ± 1.20)^b, c^	23 (2.0 ± 0.44)	
VOT	165 (24.8 ± 1.03)^c^	170 (25.6 ± 1.82)	50 (7.5 ± 0.87)^a, b^	15 (2.2 ± 0.56)	
VOT^+ ^	190 (30.4 ± 1.38)^b^	153 (24.4 ± 1.57)	57 (9.1 ± 0.51)^a^	8 (2.2 ± 0.36)	


FOT; Fresh ovaries were immediately transplanted into the testicular tissue, FOT+; Fresh ovaries were exposed to the static magnetic field (SMF) for 10 minutes and then transplanted into the testicular tissue, VOT; Vitrified-warmed ovaries were transplanted into the testicular tissue, and VOT+; Vitrified-warmed ovaries were transplanted into the testicular tissue and the transplantation site was then exposed to an SMF for 10 minutes. Values within a column with similar superscripts are not significant (P<0.05).

**Table 2:** Number of morphologically dead follicles (mean ± SEM) at different developmental stages 21 days after transplantation


Experimental group n=5	Primordial folliclen (Mean% ± SEM)	Primary folliclen (Mean% ± SEM)	Preantral folliclen (Mean% ± SEM)	Antral folliclen (Mean% ± SEM)

FOT	157 (12.8 ± 4.14)	156 (12.4 ± 4.20)	42 (3.3 ± 2.55)^b^	21 (1.7 ± 1.55)
FOT^+ ^	48 (4.11 ± 2.88)^b^	141 (12.2 ± 3.90)	45 (3.9 ± 2.44)^b^	14 (1.2 ± 1.77)
VOT	87 (13.37 ± 3.72)	116 (17.4 ± 2.43)^a^	42 (6.3 ± 2.78)^a^	18 (2.7 ± 1.47)
VOT^+ ^	77 (12.3 ± 2.74)	78 (12.4 ± 2.86)	48 (7.6 ± 1.33)^a^	8 (1.2 ± 0.90)


FOT; Fresh ovaries were immediately transplanted into the testicular tissue, FOT+; Fresh ovaries were exposed to the static magnetic field (SMF) for 10 minutes and then transplanted into the testicular tissue, VOT; Vitrified-warmed ovaries were transplanted into the testicular tissue, and VOT+; Vitrified-warmed ovaries were transplanted into the testicular tissue and the transplantation site was then exposed to an SMF for 10 minutes. Values within a column with similar superscripts are not significant (P<0.05).

### Quantitative study of angiogenesis

**Fig.3:**
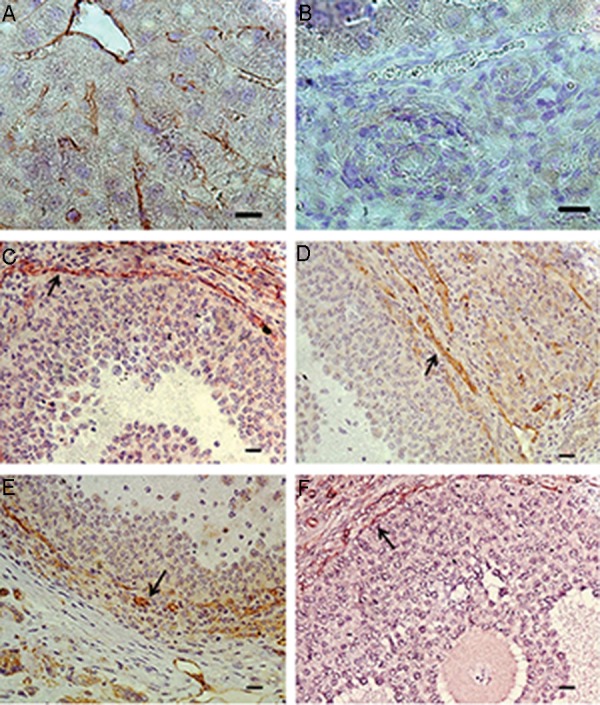
Immunohistochemical analysis for blood vessels (using the marker CD31) in the ovarian tissue transplanted into the testis. The liver tissue was used as A. Positive and B. Negative controls for CD-31, C. Fresh ovaries were immediately transplanted into the testicular tissue (FOT group), D. Fresh ovaries were exposed to the magnetic field for 10 minutes and then transplanted into the testicular tissue (FOT+ group), E. Vitrified-warmed ovaries were transplanted into the testicular tissue (VOT group), and F. Vitrified-warmed ovaries were transplanted into the testicular tissue and the transplantation site was then exposed to a magnetic field for 10 minutes (VOT+ group). Black arrows indicate blood vessels (scale bars: 10 μm).

To estimate the effect of SMF on neoangiogenesis, CD31 was identified in the transplanted ovarian tissues. CD31 or platelet endothelial cell adhesion molecule-1 (a 130-kd membrane glycoprotein of the immunoglobulin superfamily) has been identified as vascular cellspecific cell-cell adhesion molecules. It localize to endothelial cell intercellular junctions. There was a clear difference in the alteration in the number of blood vessels between the FOT+ (18.60 ± 0/51) and VOT (7.80 ± 0/58) groups, but the presence of blood vessels showed no significant difference between the other groups (Table 3, Fig .3).

### Prevalence of programmed cell death

**Fig.4:**
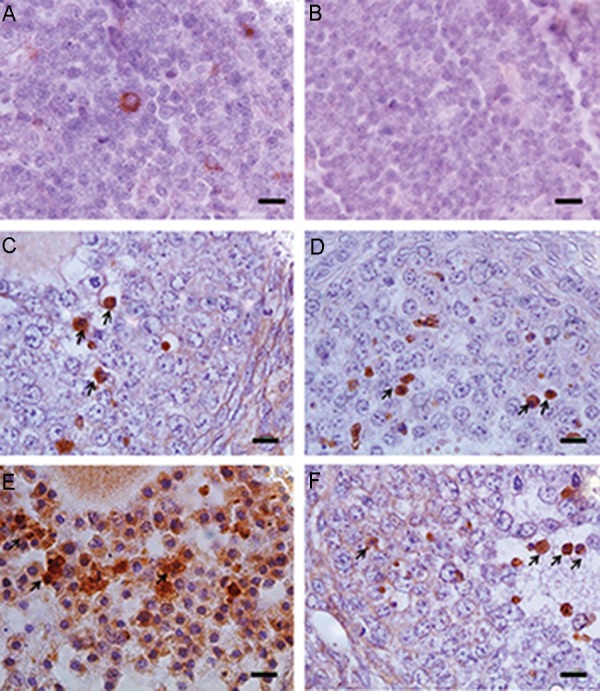
Immunohistochemical analysis for caspase-3 in the ovarian tissue transplanted into the testis. The thymus tissue was used as A. Positive, B. Negative controls for caspase-3, C. Fresh ovaries were immediately transplanted into the testicular tissue (FOT group), D. Fresh ovaries were exposed to the magnetic field for 10 minutes and then transplanted into the testicular tissue (FOT+ group), E. Vitrified-warmed ovaries were transplanted into the testicular tissue (VOT group), and F. Vitrified-warmed ovaries were transplanted into the testicular tissue and the transplantation site was then exposed to a magnetic field for 10 minutes (VOT+ group). Positive staining is shown as brown coloration of the cytoplasm of the cells, and white arrow indicates caspase-3 staining (scale bars: 10 μm).

Expression of the caspase-3 protein was not observed in the cells of surface epithelium and primordial and primary follicles, but several apoptotic caspase-3-positive cells were detected in the degenerating corpus luteum (Fig .4). In the preantral and antral follicles, the apoptosis incidence was different between the VOT (4.20% ± 0.66 and 2.20% ± 0.37) and the other groups, viz. the FOT (1.40% ± 0.24 and 0.8% ± 0.24), FOT+ (1.20% ± 0.20 and 0.60% ± 0.24) and VOT+ (2.20% ± 0.37 and 1.00% ± 0.31) groups (Table 4).

**Table 3:** New vascularisation based on CD31 expression in the transplanted tissue 3 weeks after transplantation


Experimental group n=5	Number of blood vessels(Mean% ± SEM)

FOT	14.00 ± 0.70^a, b^
FOT^+ ^	18.60 ± 0.51^a^
VOT	7.80 ± 0.58^b^
VOT^+ ^	12.20 ± 0.86^a, b^


FOT; Fresh ovaries were immediately transplanted into the testicular tissue, FOT+; Fresh ovaries were exposed to the static magnetic field (SMF) for 10 minutes and then transplanted into the testicular tissue, VOT; Vitrified-warmed ovaries were transplanted into the testicular tissue, and VOT+; Vitrified-warmed ovaries were transplanted into the testicular tissue and the transplantation site was then exposed to an SMF for 10 minutes. Values within a column with similar superscripts are not significant (P<0.05).

**Table 4:** Mean percentages of apoptotic follicles (%) at different developmental stages in transplanted mouse ovaries into testes


Experimental groupn=5	Total of folliclesn	Preantral follicles(Mean% ± SEM)	Antral follicles(Mean% ± SEM)

FOT	142	1.40 ± 0.24	0.8 ± 0.24
FOT^+^	149	1.20 ± 0.20	0.60 ± 0.24
VOT	71	4.20 ± 0.66^a^	2.20 ± 0.37^a^
VOT^+ ^	89	2.20 ± 0.37	1.00 ± 0.31


FOT; Fresh ovaries were immediately transplanted into the testicular tissue, FOT+; Fresh ovaries were exposed to the static magnetic field (SMF) for 10 minutes and then transplanted into the testicular tissue, VOT; Vitrified-warmed ovaries were transplanted into the testicular tissue, and VOT+; Vitrified-warmed ovaries were transplanted into the testicular tissue and the transplantation site was then exposed to an SMF for 10 minutes. Values within a column with similar superscripts are not significant (P<0.05).

### Hormonal assay

In grafted male mice, E_2_ and P_4_ concentrations were significantly higher than those in control mice. The experimental and control groups showed notable differences in the plasma T4 level. The control group showed the highest level of T4 (32.71 ± 0.95), which was significant compared with those of the other groups. In addition, the highest and lowest levels of T4 were observed in the FOT+ (18.20 ± 0.58) and VOT (10.60 ± 0.74) groups, respectively, but these differences were not significant (Table 5). However, the VOT group (0.04 ± 0.00) had the lowest testis weight 3 weeks after transplantation, which was comparable to the other control and experimental groups.

### *In vitro* maturation, fertilization and embryo development

**Fig.5:**
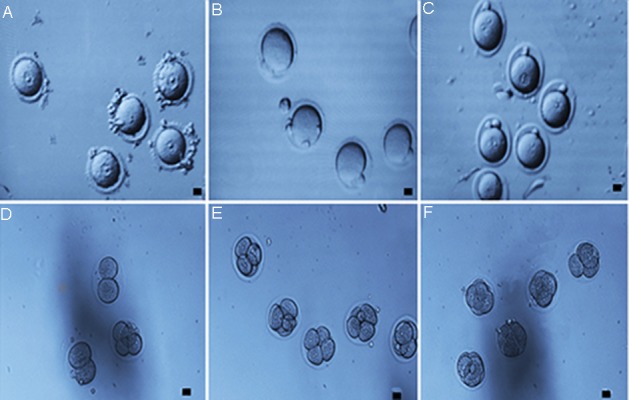
*In vitro* maturation, fertilization and cleavage of mouse germinal vesicle (GV) oocytes obtained from the ovarian tissue transplanted into the testis. A. Mouse GV oocyte, B. Oocytes extruded a polar body after overnight *in vitro* maturation, C. Fertilised 2-pronuclear zygote, and D-F. Development of oocytes derived from *in vitro* culturing after fertilization (scale bars: 10 μm).

Progression to MII stage was significantly higher in oocytes derived from the FOT+ group (51.54% ± 2.87) than in those derived from the other groups, while VOT group showed the lowest comparative MΠ rate and the highest total degeneration rate (14.28% ± 2.88 and 33.88% ± 3.47, respectively) (Table 6). The fertility rate in the VOT group (30.00% ± 13.33) was significantly different from the FOT and FOT+ groups (61.16% ± 5.68 and 69.83% ± 4.71, respectively). There was no significant difference between the VOT+ (51.66% ± 3.88) and the other groups (Table 7) for the fertility rate. The rates of formation of 2, 4 and 8-cell embryos in the VOT group (30.00% ± 15.27, 10.00% ± 12.47 and 10.00% ± 12.47, respectively) were significantly lower than those in the other groups. In addition, there was a significant difference in the embryos that reached the morula stage between the FOT+ (35.83% ± 4.97), VOT (0.00% ± 7.63) and VOT+ groups (15.00% ± 0.00) (Table 8, Fig .5).

**Table 5:** Steroid hormones changes in male mice 21 days after ovarian transplantation


Experimental groupn=5	Testis weight (g)(Mean% ± SEM)	Estrogen (pmol/l)(Mean% ± SEM)	Progesterone (nmol/l)(Mean% ± SEM)	Testosterone (nmol/l)(Mean% ± SEM)

Control	0.135 ± 0.01	368.17 ± 26.14^c^	2.1 ± 5.42^c^	32.71 ± 0.95^a^
FOT	0.100 ± 0.00	571.60 ± 18.23^a, b^	73.40 ± 7.08^b^	14.40 ± 0.60
FOT^+ ^	0.122 ± 0.00	628.40 ± 30.06^a^	114.80 ± 6.21^a^	18.20 ± 0.58
VOT	0.04 ± 0.00^b^	495.73 ± 20.43^b^	41.43 ± 3.23^b^	10.60 ± 0.74
VOT^ +^	0.09 ± 0.01	537.20 ± 30.19^a, b^	59.60 ± 10.48^b^	13.20 ± 1.06


FOT; Fresh ovaries were immediately transplanted into the testicular tissue, FOT+; Fresh ovaries were exposed to the static magnetic field (SMF) for 10 minutes and then transplanted into the testicular tissue, VOT; Vitrified-warmed ovaries were transplanted into the testicular tissue, and VOT+; Vitrified-warmed ovaries were transplanted into the testicular tissue and the transplantation site was then exposed to an SMF for 10 minutes, n; Number of mice in each experimental group. Values within a column with similar superscripts are not significant (P<0.01).

**Table 6:** Maturation of mouse oocytes recovered from the transplanted ovarian tissue into the testis after 24 hours culture *in vitro*


Experimental group n=5	Number of oocytesrecovered	Number of GV (Mean% ± SEM)	Number of GVBD(Mean% ± SEM)	Number of MΠ(Mean% ± SEM)	Number of degeneration(Mean% ± SEM)

FOT	70	10 (14.80 ± 2.01)^b^	21 (29.88 ± 4.24)	31(43.60 ± 2.51)^b^	8 (11.70 ± 2.90)
FOT^+ ^	79	12 (14.71 ± 2.26)^b^	17 (22.53 ± 3.38)	41 (51.54 ± 2.87)^a^	9 (11.20 ± 1.98)
VOT	67	18 (27.80 ± 3.44)^a^	16 (24.02 ± 3.04)	10 (14.28 ± 2.88)^c^	23 (33.88 ± 3.47)^a^
VOT^+ ^	68	16 (23.48 ± 3.00)^a^	13 (19.79 ± 2.73)	27 (39.59 ± 2.22)^b^	12 (17.11 ± 2.38)


FOT; Fresh ovaries were immediately transplanted into the testicular tissue, FOT+; Fresh ovaries were exposed to the SMF for 10 minutes and then transplanted into the testicular tissue, VOT; Vitrified-warmed ovaries were transplanted into the testicular tissue, VOT+; Vitrified-warmed ovaries were transplanted into the testicular tissue and the transplantation site was then exposed to an SMF for 10 minutes, SMF; Static magnetic field, GV; Germinal vesicle, GVBD; GV breakdown, and MΠ; Metaphase II. Values within a column with similar superscripts are not significant (P<0.05).

**Table 7:** Comparison of *in vitro* fertilization rate between the experimental groups


Experimental group	Number of MΠ	Number of 2PN(Mean% ± SEM)

FOT	31	19 (61.29 ± 5.68)^a^
FOT^+^	41	28 (68.29 ± 4.71)^a^
VOT	10	3 (30.00 ± 13.33)^b^
VOT^+^	27	14 (51.85 ± 3.88)^a, b^


FOT; Fresh ovaries were immediately transplanted into the testicular tissue, FOT+; Fresh ovaries were exposed to the static magnetic field (SMF) for 10 minutes and then transplanted into the testicular tissue, VOT; Vitrified-warmed ovaries were transplanted into the testicular tissue, VOT+; Vitrifiedwarmed ovaries were transplanted into the testicular tissue and the transplantation site was then exposed to an SMF for 10 minutes, 2PN; 2 pronuclei, and MΠ; Metaphase II. Data are presented as mean percent ± SEM. Values within a column with similar superscripts are not significant (P<0.05).

**Table 8:** *In vitro* development of embryos after *in vitro* fertilization


Experimental group	Total	2-cell (%)	4-cell (%)	8-cell (%)	Morula (%)

FOT	70	91.66 ± 5.69	66.66 ± 10.82	55.00 ± 10.84	20.00 ± 6.93^a, b^
FOT^+^	79	92.50 ± 5.33	70.00 ± 5.85	55.55 ± 4.84	35.83 ± 4.97^a^
VOT	67	30.00 ± 15.27^b^	10.00 ± 12.47^b^	10.00 ± 12.47^b^	0.00 ± 7.63^c^
VOT^+^	68	80.00 ± 13.33	60.00 ± 10.00	40.00 ± 10.00	15.00 ± 0.00^b, c^


FOT; Fresh ovaries were immediately transplanted into the testicular tissue, FOT+; Fresh ovaries were exposed to the static magnetic field (SMF) for 10 minutes and then transplanted into the testicular tissue, VOT; Vitrified-warmed ovaries were transplanted into the testicular tissue, and VOT+; Vitrified-warmed ovaries were transplanted into the testicular tissue and the transplantation site was then exposed to an SMF for 10 minutes. Data are presented as mean percent ± SEM. Values within a column with similar superscripts are not significant (P<0.05).

## Discussion

In the current study, the testis was used as an alternative site for ovarian transplantation. Testis has been recognised as an immunologically privileged site that can enhance graft acceptance (20). Studies have shown that Sertoli cells of the testis express Fas ligand, which prevents graft rejection (21). We therefore transplanted the ovary into the testis without immunosuppression in male mice to determine whether the testis provides a unique environment for suppressing immune responses and improving graft acceptance. The study findings indicate that the environment of adult testis promote normal development of the follicles. These results are consistent with the findings of Sato et al. (22), but differ from those of a previous study (23).

In addition, we studied the effect of SMFs on ovarian transplantation; here we focused on the study of the magnetic field itself, and to the best of our knowledge, there have been no prior studies on the effect of SMF on reproductive organ transplantation. SMFs are generally classified on the basis of intensity as weak (<1 mT), moderate (1 mT to 1 T), strong (1-5 T), and ultrastrong (>5 T) (24). SMFs with moderate intensity have been shown to be effective on biological systems (25). Magnetic field therapy by means of moderateintensity SMF could be beneficial for circulatory diseases, including ischemic pain, inflammation and hypertension, because of the modulation of blood flow and/or blood pressure (26). Hence, in this study, SMF with moderate intensity was used during ovarian transplantation. The study results showed the highest preservation of primordial follicles in the group where fresh ovarian tissue was exposed to the SMF before transplantation. Healthier primordial follicles were seen in the group of transplanted vitrifiedwarmed ovaries (VOT+), when the transplantation area was exposed to SMF, than in the VOT group that was not subjected to SMF before and after transplantation. It seems that using SMF during ovarian transplantation can exert positive effects and lead to better retention of ovarian follicles after transplantation. An interesting observation during histological evaluation was that in the FOT+ and VOT+ groups, it seemed that the testis was able to produce sperm, even when follicles of the transplanted ovarian tissue inside it were growing and developing. In tissue sections, follicles with good quality at different growth stages and corpus luteum were observed, and blood vessels around the grafts were clearly seen in all experimental groups. However, in some transplanted ovaries, injuries such as altered normal morphology and oocyte degeneration were observed. These changes in the VOT group were more specific. Although a weak inflammation sign was observed in all the experimental groups, the presence of large granular lymphocytes and the resident macrophages was not clear in tissue sections. Griffith et al. (27) reported that the expression of FasL in the testis can limit inflammation reactions and confer immunoprivilege by allowing them to kill infiltrating lymphocytes expressing Fas. In addition, activated lymphocytes that co-express Fas and FasL become susceptible to apoptosis.

A previous study suggested that the rapid establishment of a rich blood supply is one of the most essential factors for successful ovarian tissue transplantation and survival of the ovarian follicles (28). We assessed angiogenesis in the grafts by anti-mouse CD31 immunohistochemical staining. Many newly formed blood vessels were observed in the ovaries exposed to the SMF before transplantation (although not statistically significant). The smallest number of blood vessels was seen in the VOT as compared to the FOT+ group. It has been reported that SMFs affect vessel growth and development both *in vitro* and *in vivo* (29). Similarly, Bassett showed that local exposure to SMF leads to enhanced angiogenesis and ossification in bone (30). It was also reported that electromagnetic fields increase *in vitro* and *in vivo* angiogenesis through endothelial release of FGF-2 (31). In the present study, the SMF did not reduce the number of blood vessels and angiogenesis.

In some studies, correlation was observed between increments in ovarian angiogenesis with reduction in tissue hypoxia and programmed cell death of follicles (32). In the previous reports, it is stated that magnetic fields may delay cell death caused by ischemia and reduce the size of the ischemic penumbra by improving collateral blood flow to the ischemic area (33). Fanelli et al. (34) suggested that magnetic fields increase cell survival by inhibiting apoptosis through modulation of Ca^2+^ influx. In contrast, some other researchers believe that magnetic field increases apoptosis (35). The extent of this effect seems to depend on the cell type, intensity and duration of the radiation field; permeability of tissues; and other experimental conditions (36, 37).

In current study, we used caspase-3 as an apoptosis marker, which in its active form is used as a marker for apoptotic death in the early stages of apoptosis (38, 39). A stained reddish brown colour of the cytoplasm/nucleus of the follicles was considered as positive (39). Evaluation of apoptotic incidence on mouse ovaries did not present any caspase-3 positive follicles in the stages of primordial and primary. These results are contrary to previous research which reported caspase-3 positive follicles in the stages of primordial and primary (18).

The study result showed that SMF with 1 mT intensity does not increase apoptosis in ovarian follicles. An improved follicular survival with no increase in apoptosis was observed when the ovarian tissues were exposed to SMF before transplantation into the testes. In preantral and antral follicles, after transplantation, the rate of programmed cell death in the VOT group was significantly higher than those in the other groups. It should be noted that in the VOT group, a lower angiogenesis rate was observed. Despite the fact that in some cases, the differences were not statistically significant between the groups, our study results showed an association between the increase in angiogenesis and apoptosis reduction.

The study results demonstrate that the use of SMF after ovarian tissue transplantation of vitrifiedwarmed ovaries significantly improved the quality of transplanted ovarian tissues by decreasing apoptosis. These results are confirmed by previous studies (34). For the fresh transplanted groups, the difference was not statistically significant in terms of programmed cell death, but there was an obvious reduction in apoptosis in the FOT+ group. However, the exact mechanism of the beneficial effect of SMF is uncertain.

To understand follicular and oocyte development, we investigated the endocrine function of grafts in male mice receiving ovarian tissues. The plasma concentrations of E_2_ and P_4_ hormones in male mice with ovarian transplants were significantly higher than those in mice without ovarian transplants. These data indicate hormone secretion by the follicles in the grafted ovaries into the testes. These results are similar to the results of Li et al. (40).

The findings showed that follicles in grafted ovarian tissue (both fresh and vitrified-warmed) developed and the grafts had ample blood vessels. One of the most important concerns about ovarian transplantation into the testis is whether the implant can ovulate oocytes in response to exogenous gonadotropins (22); to address this concern, we studied ovarian graft functionality by retrieving oocytes for *in vitro* maturation followed by *in vitro* fertilization by mature epididymal spermatozoa. The rate of oocytes reaching MII stage was higher in the FOT+ than in the other experimental groups, whereas it was lowest in the VOT group. A higher percentage of degenerated oocytes, low *in vitro* fertilization and embryo development rates were observed in the VOT group. The only difference between the VOT and VOT+ groups was that the application of a magnetic field after transplantation in the VOT+ group exerts positive effects on embryo development. In this study, an SMF was applied during ovarian vitrification and transplantation. During vitrification, the ovarian tissues were exposed to a magnetic field for 15 minutes in the equilibration step. After thawing, vitrified-warmed tissues were transplanted into male mice (VOT group). The apoptosis rate, oocyte maturation, fertilization and *in vitro* embryonic development of the FOT and VOT groups were significantly different from each other. It was therefore decided to determine the effect of SMF on vitrified-warmed ovaries transplantation. For this, vitrified-warmed ovaries were transplanted into the testicular tissue and the transplantation site was then exposed to a magnetic field for 10 minutes (VOT+ group). The data showed that the use of SMF after vitrifiedwarmed ovaries transplantation significantly improved the maturation rate and embryonic development (except morula). It also reduced the apoptosis rate.

## Conclusion

The testis can provide an environment for improving ovarian graft acceptance. SMF coupled with transplantation procedure increases the survival rates of grafted ovarian follicles. In addition, exposure of the vitrified-warmed ovaries to SMF retains the structure of the graft similar to that of fresh ovaries. Further studies are necessary Ovarian Tissue Transplantation under Influence of SMF to determine the exact mechanism of SMF effects on ovarian transplantation.
